# Clinical Risk Factors and First Gestational 75 g OGTT May Predict Recurrent and New-Onset Gestational Diabetes in Multiparous Women

**DOI:** 10.3390/jcm13175200

**Published:** 2024-09-02

**Authors:** Maria Mirabelli, Vera Tocci, Eusebio Chiefari, Stefano Iuliano, Francesco S. Brunetti, Roberta Misiti, Stefania Giuliano, Marta Greco, Daniela P. Foti, Antonio Brunetti

**Affiliations:** 1 Department of Health Sciences, University “Magna Græcia” of Catanzaro, 88100 Catanzaro, Italy; maria.mirabelli@unicz.it (M.M.); tocci.vera@gmail.com (V.T.); marta.greco@unicz.it (M.G.); 2 Operative Unit of Endocrinology, “Renato Dulbecco” University Hospital, 88100 Catanzaro, Italy; stefania.giuliano75@gmail.com; 3 Operative Unit of Clinical Pathology, “Renato Dulbecco” Hospital, 88100 Catanzaro, Italy; 4 Department of Experimental and Clinical Medicine, University “Magna Græcia” of Catanzaro, 88100 Catanzaro, Italy

**Keywords:** gestational diabetes, recurrence, oral glucose tolerance test, IADPSG, risk factors

## Abstract

**Background**: Women who experience gestational diabetes mellitus (GDM) during their first pregnancy are at a high risk of developing GDM again in subsequent pregnancies. Even mothers with no previous history of GDM may develop the condition in a new pregnancy. **Methods**: In this retrospective cross-sectional observational study, 759 multiparous women tested for GDM in two successive pregnancies using the 75 g OGTT (IADPSG criteria) were enrolled. The OGTT was performed at 24–28 weeks’ gestation or earlier if there was a history of GDM. Participants were categorized into four groups: women with normal glucose tolerance (NGT) in both pregnancies (*n* = 493), women with a first occurrence of GDM in their second pregnancy (*n* = 74), women with non-recurrent GDM in their second pregnancy (*n* = 92), and women with recurrent GDM in their second pregnancy (*n* = 100). **Results**: Intergroup comparisons revealed clinical predictors of GDM in the first pregnancy (family history of type 2 diabetes, PCOS, advanced maternal age, pregravid obesity) and in the second pregnancy (interpregnancy BMI gain), as well as predictors of recurrent GDM (pregravid obesity, PCOS). A positive correlation was observed between the OGTT glucose levels of consecutive pregnancies. Adjusted logistic regression indicated that a higher 1-h post-load glucose level (≥130 mg/dL) during the first pregnancy significantly increased the likelihood of new-onset GDM in the second pregnancy (OR: 2.496), whereas a higher 2-h post-load glucose level (≥153 mg/dL) at the first diagnostic OGTT increased the likelihood of recurrent GDM (OR: 2.214). **Conclusions**: Clinical risk factors and post-load glucose levels during the first gestational 75 g OGTT can help predict new-onset or recurrent GDM in multiparous women.

## 1. Introduction

Clinical studies consistently demonstrate that women who develop gestational diabetes mellitus (GDM) during pregnancy are at high risk of experiencing GDM in subsequent pregnancies [1,2,3,4]. The highest recurrence rates are observed using the International Association of the Diabetes and Pregnancy Study Groups (IADPSG) criteria, which are designed to detect increased risk for several adverse pregnancy outcomes for both mothers and children [5]. Ethnicity also influences recurrence rates, with non-Hispanic white women showing lower rates (39%) compared to women of other ethnic backgrounds (56%). Primiparous women have lower rates of GDM (40%) compared to multiparous women (73%) [6]. The increased prevalence of GDM in multiparous women is linked to physiological mechanisms involving impairments in insulin action and beta-cell function. Although many women return to normal glucose tolerance after delivery, persistent subtle defects in glucose metabolism contribute to GDM recurrence in subsequent pregnancies and increase the risk of developing postpartum type 2 diabetes (T2D) [7,8,9].

The risk factors for GDM recurrence can be categorized into unmodifiable factors (such as patient age, number of previous pregnancies, family history of diabetes, and insulin use during a previous pregnancy complicated by GDM) and modifiable factors (such as time interval and weight gain between successive pregnancies, pregravid BMI, and high fasting glucose levels in the first trimester of pregnancy). A recent study conducted in China from 2016 to 2019 uncovered significant differences in the risk factors for GDM between women with a previous GDM diagnosis and those without. Among women with multiple pregnancies but no prior history of GDM, new-onset GDM was linked to advanced maternal age, higher pregravid BMI, longer intervals between pregnancies, family history of T2D, and elevated levels of glucose and lipids in early pregnancy [5]. Conversely, recurrent GDM was associated with excessive weight gain during pregnancy, increased BMI, and higher fasting glucose levels in subsequent pregnancies [5]. Conditions such as higher BMI in subsequent pregnancies and weight gain between pregnancies were identified as contributors to GDM recurrence [2]. Supporting the impact of interpregnancy weight changes on GDM risk, population-based cohort studies indicate that weight loss between pregnancies, especially among overweight or obese women with a history of GDM, significantly reduces the incidence of GDM recurrence [10]. This weight loss enhances peripheral insulin sensitivity, thereby diminishing the risk of GDM and associated complications. Encouraging women with GDM to pursue postpartum weight loss, aiming for a reduction of ≥5% before planning a new pregnancy, has demonstrated an 82% decrease in the likelihood of GDM recurrence [11]. This strategy not only mitigates GDM risk but also potentially lowers the chances of developing T2D in the future [11].

In this study, we investigated the clinical and laboratory risk factors contributing to both GDM recurrence and new-onset in a contemporary cohort of Mediterranean women from the Calabria Region of Southern Italy. Metabolic disturbances during gestation, as defined by the IADPSG criteria, are particularly prevalent in this population and significantly impact public health due to their long- and short-term consequences for both mothers and children [12,13]. Understanding the predictors of GDM in women intending to have multiple pregnancies is crucial for effective pregnancy planning and for avoiding the transgenerational effects of GDM.

## 2. Materials and Methods

### 2.1. Study Population

This monocentric, retrospective cross-sectional observational study included 759 consecutive Caucasian women with two sequential singleton pregnancies (gestation duration > 24 weeks) who attended the tertiary care Endocrinology Unit of “Renato Dulbecco” Hospital in Catanzaro, Italy, from January 2013 to March 2020. The study was completed before the onset of the COVID-19 pandemic, which later led to temporary changes in screening practices to reduce hospital visits and limit virus exposure [14]. During the study period, GDM screening was conducted using a 75 g oral glucose tolerance test (OGTT), following the criteria set by the IADPSG and the Italian “Istituto Superiore di Sanità” (ISS) guidelines, which have been in effect since 2010 [15].

In Italy, early GDM screening at 16–18 weeks of gestation is recommended for women with significant risk factors such as pregravid obesity, impaired fasting glucose (IFG) in the pregravid period or first trimester, or a history of GDM. If early screening results for these high-risk women are negative, a repeat 75 g OGTT is advised between 24 and 28 weeks of gestation [15]. However, despite the ISS guidelines suggesting a two-step, risk-factor-based approach, in practice, most women undergo the 75 g OGTT at 24–28 weeks regardless of risk factors, with early screening often disregarded in the absence of prior GDM [16,17]. For this study, GDM was diagnosed if any venous plasma glucose values exceeded IADPSG thresholds: fasting ≥ 92 mg/dL, 1-h post-load ≥ 180 mg/dL, or 2-h post-load ≥ 153 mg/dL. Blood samples were processed within 30 min in an on-site clinical laboratory, with glucose levels measured using the enzymatic glucose oxidase method on the ILab650 chemistry analyzer (Instrumentation Laboratory, Werfen LLC, Bedford, MA, USA). The coefficient of variation was 1.5% at a mean glucose concentration of 73 mg/dL and 0.9% at 248 mg/dL, meeting the recommended analytical precision standards. Maternal demographic and clinical data, representing potential risk factors for GDM, including age, education level, family history of T2D, previous GDM, parity, reproductive history, age of menarche, polycystic ovary syndrome (PCOS), smoking status, last menstrual period, and pregravid body weight, were recorded in a digital patient diary (Smart Digital Clinic^®^, Meteda Srl, San Benedetto del Tronto, Italy). Core anthropometric measurements were collected during the 75 g OGTT. Pregravid BMI and gestational weight gain before the screening test were calculated, with BMI defined as weight divided by height squared (kg/m^2^). Gestational weight gain was determined as the difference between body weight at the 75 g OGTT and pregravid body weight. For those diagnosed with GDM and under clinical observation at our institution, gestational weight gain was also measured at the last follow-up before delivery (within 3 weeks). Exclusion criteria included twin pregnancies, chronic systemic diseases, or medications affecting glucose tolerance (e.g., metformin [18]). Data on macrosomia (neonatal birthweight ≥ 4 kg) and preterm birth (before 37 weeks) from the index pregnancy were also collected. For most participants, the index pregnancy, defined as the first of the two sequential pregnancies examined in the study, also corresponded to their first childbirth experience. Hence, the terms “first pregnancy” and “index pregnancy” are used interchangeably in the following sections of the manuscript.

### 2.2. Statistical Analysis

Continuous variables are expressed as medians and interquartile ranges (IQRs), while categorical variables are expressed as numbers and percentages. The Mann–Whitney U test was used to compare continuous variables between women with and without GDM. Fisher’s exact test was used for comparing proportions. Spearman’s correlation tests assessed the univariate relationship between venous plasma glucose values at the 75 g OGTT during the index and subsequent pregnancies. A heat map of correlation coefficients was generated. Logistic regression models were employed to confirm the influence of 75 g OGTT glucose values at the index pregnancy on the likelihood of GDM diagnosis in a subsequent pregnancy, controlling for potential confounders. Adjusted odds ratios (OR) with 95% confidence intervals were calculated. A significance level of 0.05 was set for all analyses, which were conducted using JASP Graphical Statistical Software Version 0.17.1.0 (University of Amsterdam, Amsterdam, The Netherlands) based on R Stats packages.

## 3. Results

### 3.1. Exploring Risk Factors for GDM through Retrospective Digital Health Data Collection

Out of the 759 multiparous women enrolled, 192 (25.3%) were diagnosed with GDM during their first pregnancy, consistent with the high prevalence rates of this condition reported in Southern Italy since the implementation of the IADPSG criteria. The remaining 567 women, who had normal glucose tolerance (NGT) in their first pregnancy, were split into Group 1 (493 women, 87.01%) with no GDM in either pregnancy and Group 2 (74 women, 13.05%) with new onset GDM in the subsequent pregnancy, supporting the association between increased GDM risk, parity and advancing maternal age. The 192 women with GDM in the first pregnancy were divided into Group 3 (92 women, 47.92%) with no recurrence, and Group 4 (100 women, 52.08%) with recurrent GDM, confirming the frequent tendency for women with GDM to experience the same diagnosis in a subsequent pregnancy. Clinical and laboratory characteristics, as well as data on macrosomia and preterm birth from the first pregnancy, are detailed in Table 1. Intergroup comparisons revealed that women with GDM in the first pregnancy were older (median age: 31 vs. 30 years, *p* = 0.003) and had higher pregravid BMI (Group 4 vs. Group 1, median BMI 26.0 vs. 22.3, *p* < 0.001). Pregravid obesity was more common among women with GDM at the first pregnancy and with GDM recurrence. A family history of T2D was also more frequent in women with GDM, and PCOS was more prevalent (1.6% and 2.7% in Groups 1 and 2 vs. 10.9% and 34.0% in Groups 3 and 4, *p* < 0.001). Group 2 women, who developed GDM in the second pregnancy, had higher pregravid BMI (24.4 vs. 22.9, *p* = 0.024) and higher OGTT glucose levels in both pregnancies, especially 1-h post-load values during the first pregnancy (133.5 vs. 122.0 mg/dL, *p* < 0.001) compared to persistently NGT women. Women with recurrent GDM had higher pregravid BMI before the second pregnancy, tended to gain more weight during the interpregnancy period, and more often required insulin therapy during the first pregnancy due to more severe glycemic decompensation (42.3% vs. 10.0%, *p* = 0.012), as indicated by higher 75 g OGTT glucose levels, especially 1-h and 2-h post-load (median AUC glucose: 302.0 vs. 287.8 mg·h/dL, *p* = 0.028). Overall, this finding underscores the importance of elevated post-load glucose values in identifying β-cell dysfunction, insulin resistance, and a more adverse metabolic risk profile in pregnant women, consistent with findings in non-pregnant populations.

### 3.2. Correlations between First and Second Gestational 75 g OGTTs

**Figure 1:**
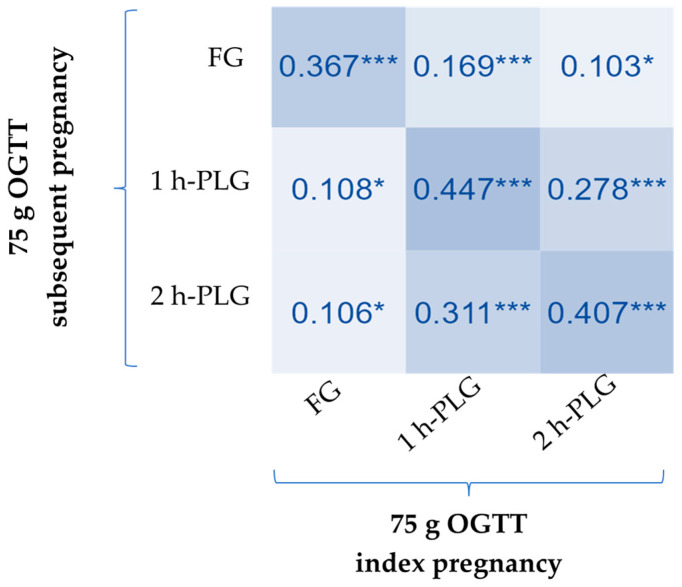
Heat map of Spearman’s correlation coefficients. *** indicates statistical significance with *p* < 0.001. * indicates statistical significance with *p* < 0.05. FG: fasting glucose; PLG: post-load glucose.

We aimed to mitigate potential biases stemming from inter-laboratory analytical differences by enrolling women who underwent the gestational 75 g OGTT at the same diabetes care facility for both of their successive pregnancies. Venous blood samples were promptly processed in an on-site clinical laboratory specializing in diabetes care, using the same chemistry analyzer. This consistency allowed for a precise evaluation of the correlation between glucose levels measured during the 75 g OGTT in the first and second pregnancies. Our analysis, illustrated in Figure 1, demonstrated moderate positive correlations between the 1-h and 2-h post-load glucose levels of consecutive pregnancies, supported by Spearman’s coefficients > 0.4.

### 3.3. Regression and Receiving Operating Characteristic (ROC) Analysis to Test the Predictive Effects of First Gestational 75 g OGTT Post-Load Glucose Values

**Figure 2:**
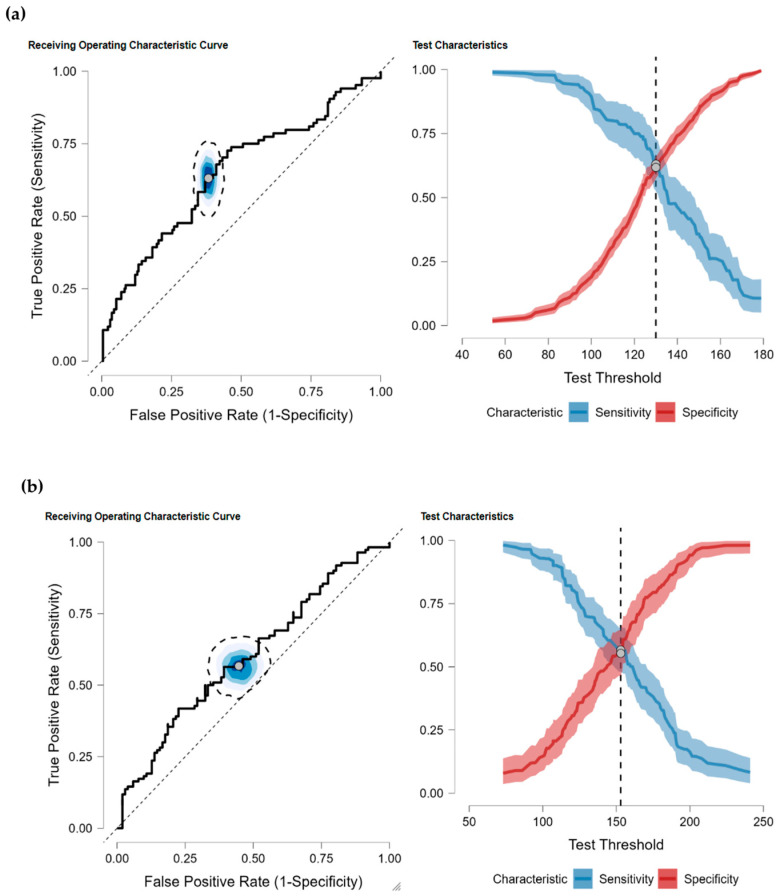
ROC analysis for determining optimal predictive thresholds of 1-h and 2-h post-load glucose values from the first gestational 75 g OGTT to identify (**a**) new-onset GDM or (**b**) GDM recurrence in a subsequent pregnancy. The sensitivity and specificity characteristics for each threshold are illustrated, with sensitivity represented by a blue line and specificity by a red line.

Then, we verified the independent effects of 1-h and 2-h post-load glucose values derived from the first gestational 75 g OGTT in predicting either new onset GDM or recurrent GDM in subsequent pregnancies, employing logistic regression analysis while adjusting for potential confounders (Table 2). As a further step, to ascertain the optimal predictive thresholds of post-load glucose values from the first gestational 75 g OGTT, we performed ROC analysis (illustrated in Figure 2).

The findings of ROC analysis, supported by adjusted logistic regression results (presented in Table 3), revealed that a 1-h post-load glucose level of ≥130 mg/dL can predict the development of GDM in a subsequent pregnancy for women with NGT, while a 2-h post-load glucose level of ≥153 mg/dL can predict the recurrent GDM in women previously diagnosed with the condition during their first pregnancy. Notably, this latter threshold aligns with the criteria established by the IADPSG for diagnosing GDM, suggesting that women identified with GDM based on 2-h post-load glucose levels in a first gestational 75 g OGTT are at higher risk of experiencing hyperglycemia again in a second pregnancy.

## 4. Discussion

Women who develop GDM during their first pregnancy are at high risk of experiencing GDM in subsequent pregnancies and developing postpartum T2D [1,2,3,4,5,6,7]. This increased long-term metabolic risk is due to a combination of worsening insulin sensitivity during pregnancy, an inadequate compensatory beta-cell response, and a significant reduction in beta-cell mass postpartum [19]. Additionally, women with NGT in their first pregnancy may develop GDM in later pregnancies due to aging and the emergence of new potential risk factors [20].

The OGTT is the gold standard for diagnosing diabetes because it mimics the physiological glucose and insulin dynamics seen after a meal [21]. Despite its reliability, the approach to OGTT-based diagnosis of GDM varies globally. Some countries use a two-step procedure, starting with a 50-g, 1-h glucose challenge test, followed by the administration of 75 or 100 g of glucose, with glucose levels measured at various intervals (fasting, 1-h, 2-h, or over 3-h post-load) to diagnose GDM [9]. The glucose thresholds for diagnosis and the timing of the gestational OGTT also differ between guidelines [16]. In our study of Southern Italian Caucasian women, we observed a GDM prevalence of 25.3% during the first pregnancy, markedly higher than global estimates of around 4% worldwide [22], 5% in Europe [23], and 10% in Eastern and South Eastern Asia [22]. These variances likely result from differing screening methods and diagnostic criteria over time [9,22]. The prevalence of GDM under the IADPSG criteria, applied in this study, tends to be higher [24]. Our results are, in fact, consistent with other studies in similar regions and populations, where prevalence ranged from 25.7% to 30% [25,26], underscoring the Southern Mediterranean as a high-risk area for metabolic disorders [9,20,27]. In the context of evolving diagnostic criteria for GDM and ongoing debates about the optimal procedure, the OGTT may serve not only as a diagnostic tool for detecting GDM but also for stratifying maternal metabolic risk, which is dynamic and changes over time [28]. Physiological studies indicate that pregnancy typically lowers both preprandial and postprandial blood glucose levels, resulting in minimal daily glycemic fluctuations [9]. Continuous glucose monitoring has shown that glycemic peaks occur approximately 70 min after the start of a meal and do not exceed 122 mg/dL [29]. Current recommendations for managing GDM aim to replicate the physiological gestational glucose profile as closely as possible, maintaining fasting blood glucose levels below 90 mg/dL and 1-h postprandial levels below 130 mg/dL, with minimal variations across guidelines [15,30,31].

Postprandial glucose levels, particularly within the first hour after a meal, reflect the initial phase of insulin secretion, which is often impaired in patients with GDM [32]. Elevated postprandial glucose levels are a key driver of fetal overgrowth and can cause placental damage in women with pregestational diabetes or GDM [32,33,34]. Maternal postprandial glycemia is positively correlated with resistance indices of the uterine arteries, and high postprandial glucose levels can interfere with uterine artery remodeling [33]. This interference can potentially lead to preeclampsia and, consequently, preterm birth [35]. Although our study did not include data on preeclamptic pregnancies, we observed that women with NGT in their first pregnancy who developed GDM in their second pregnancy had higher post-load glucose levels, particularly at 1-h post-load, during the 75 g OGTT in their first pregnancy. Furthermore, these women experienced significantly higher rates of preterm births compared to women who remained NGT in both pregnancies.

Postprandial hyperglycemia is one of the earliest abnormalities in glucose homeostasis in the natural course of T2D. In nonpregnant populations, 1-h post-load plasma glucose levels above 155 mg/dL are indicative of reduced beta-cell function, intermediate hyperglycemia, and a high risk of progressing to T2D. Research spanning over 40 years, as summarized in a recent position statement by the International Diabetes Federation, indicates that elevated 1-h post-load plasma glucose may more accurately predict the progression from prediabetes to T2D compared to HbA1c [36]. It also serves as a better long-term predictor of cardiovascular morbidity and mortality [37]. Combining the 1-h post-load value with fasting plasma glucose or the 2-h post-load value does not enhance its predictive capacity. Thus, in nonpregnant populations, 1-h post-load glucose is a reliable indicator of glucose homeostasis abnormalities and the need for referral to diabetes prevention programs [36].

In our study, we found that elevated 1-h post-load glucose levels above 130 mg/dL during the 75 g OGTT in the index pregnancy were positively associated with new-onset GDM in subsequent pregnancies and possibly an increased risk of progression to postpartum T2D. This novel association may be explained by physiological changes in glycemic patterns observed in pregnant women compared to their nonpregnant state. Furthermore, women with elevated post-load glucose values during the diagnostic 75 g OGTT of their first pregnancy were at a higher risk of recurrent GDM, especially those diagnosed with GDM based on 2-h post-load glucose levels exceeding IADPSG thresholds. In nonpregnant populations, high 2-h post-load glucose levels indicate prolonged glucose tolerance impairment, reflecting a later stage in the progression from prediabetes to T2D, while 1-h post-load glucose serves as an earlier marker of diabetes transition [38]. This observation aligns with our study, which found that women with recurrent GDM are at the highest long-term cardiometabolic risk within the cohort, characterized by worse glycemic status and a greater accumulation of insulin-resistance-related risk factors (e.g., pregravid obesity, PCOS).

Women with recurrent GDM should undergo intensive lifestyle modifications and interventions, including weight loss, in the postpartum period to reduce their risk of progressing to T2D, which is estimated to be three times higher than that of women with non-recurrent GDM [39]. Identifying maternal predictors for new and recurrent GDM by integrating clinical risk factors with results from the initial 75 g OGTT could enable personalized diabetes prevention strategies and help reduce the transgenerational transmission of diabetes to children born to women with GDM.

The study has several limitations, including its retrospective design and missing data on potential protective factors such as the duration of breastfeeding after the index pregnancy, which is associated with improved glucometabolic outcomes [40]. Additionally, the findings may not be generalizable to populations with lower prevalence rates of GDM, those using diagnostic criteria different from those of the IADPSG, or socioeconomic settings where women typically experience more than two childbirths during their reproductive life (e.g., Saudi Arabia [41]). However, the study’s monocentric design, homogeneous clinical assessment of women, and absence of interlaboratory variability in GDM screening procedures strengthen the analysis by allowing for accurate correlation of gestational glucose curves and minimizing bias in result interpretation.

## 5. Conclusions

We propose that integrating clinical assessments to identify risk factors with the results of the first gestational 75 g OGTT could help in identifying women at the highest risk for either new-onset GDM or recurrent GDM in future pregnancies. Specifically, women diagnosed with GDM based on elevated glucose levels after a 2-h post-load test should receive thorough counseling and targeted interventions for weight loss during the postpartum period. This approach is crucial for managing their long-term risk of diabetes, especially if they plan to conceive again.

## Figures and Tables

**Table 1:** Clinical and 75 g OGTT characteristics of women.

Clinical and 75 g OGTT Characteristics of Women	NGT at Index (or First) Pregnancy		GDM at Index (or First) Pregnancy	
	Group 1 (*n* = 493) NGT at Subsequent Pregnancy	Group 2 (*n* = 74) GDM in Subsequent Pregnancy	Group 3 (*n* = 92) Non-Recurrent GDM	Group 4 (*n* = 100) Recurrent GDM
Ethnicity	Caucasian	Caucasian	Caucasian	Caucasian
Secondary or tertiary level education (*n*)	433 (87.8%)	64 (86.4%)	84 (91.3%)	84 (84.0%)
Smoker or ex-smoker (*n*)	64 (13.0%)	10 (13.5%)	9 (9.8%)	8 (8%)
Familial history of T2D (*n*)	250 (50.7%)	50 (67.6%)	67 (72.9%) §	68 (68.0%)
Age at menarche (y)	12 (11–13)	12 (11–13)	12 (11–13)	12 (11–12) ‡•
PCOS (*n*)	8 (1.6%)	2 (2.7%)	10 (10.9%) §	34 (34.0%) ‡¤•
Nullipara women at index pregnancy (*n*)	304 (61.7%)	48 (64.9%)	65 (70.7%)	69 (69.0%)
Reproductive history of spontaneous abortion (*n*)	100 (20.3%)	10 (13.5%)	16 (17.4%)	16 (16.0%)
Pregravid body weight index pregnancy (kg)	59.0 (53.0–67.0)	61.5 (55.0–68.0)	61.0 (55.8–70.0)	68.0 (57.8–78.0) ‡¤•
Pregravid BMI index pregnancy (kg/m^2^)	22.3 (20.4–25.0)	23.4 (20.9–25.5)	22.8 (20.9–25.8)	26.0 (22.2–29.1) ‡¤•
Pregravid obesity index pregnancy (*n*)	29 (5.9%)	9 (12.2%) ¶	10 (10.9%)	17 (17.0%) ‡
Pregravid overweight index pregnancy (*n*)	95 (19.3%)	15 (20.3%)	21 (22.9%)	39 (39.0%) ‡¤•
Maternal age index pregnancy (y)	30 (27–33)	30 (27–33)	31 (28–34) §	30 (27–33)
Standard 75 g OGTT index pregnancy, gestational age (wg)	27.0 (26.0–27.0)	26.5 (26.0–27.0)	27.0 (26.0–27.0) §†	27.0 (26.0–27.0)
Standard 75 g OGTT index pregnancy, FG (mg/dL)	80.0 (76.0–84.0)	82.0 (78.0–87.8) ¶	92.0 (85.0–94.0) §†	93.00 (84.0–97.0) ‡¤•
Standard 75 g OGTT index pregnancy, 1 h-PLG (mg/dL)	122.0 (106.0–141.0)	133.5 (119.8–152.0) ¶	168.0 (144.0–191.3) §†	182.0 (162.3–192.0) ‡¤
Standard 75 g OGTT index pregnancy, 2 h-PLG (mg/dL)	100.0 (88.0–113.0)	107.5 (94.0–119.5) ¶	146.00 (120.8–168.0) §†	156.5 (123.8–182.3) ‡¤
Standard 75 g OGTT index pregnancy, AUC glucose (mg·h/dL)	213.0 (192.0–235.5)	232.3 (204.7–250.6) ¶	287.8 (255.0–317.4) §†	302.0 (275.4–323.0) ‡¤•
Standard 75 g OGTT index pregnancy, gestational weight gain (kg)	7.0 (5.0–9.0)	7.0 (5.0–9.0)	7.0 (5.0–9.0)	6.0 (3.0–8.0) ‡
Gestational weight gain at the last follow-up before delivery (kg)	-	-	10.0 (7.3–12.0)	8.0 (5.0–9.8)
Insulin therapy index pregnancy (*n*)	-	-	3 (10% *)	11 (42.3% *) •
Macrosomic birth index pregnancy (*n*)	2 (0.4%)	1 (1.4%)	0 (0%)	1 (1.0%)
Preterm birth index pregnancy (*n*)	3 (0.6%)	3 (4.1%) ¶	5 (5.4%) §	1 (1.0%)
Maternal age subsequent pregnancy (y)	33 (30–36)	33 (31–36)	34 (31–37) §	33 (31–36)
Interpregnancy age change (y)	3.0 (2.0–5.0)	4.0 (2.3–5.0)	3.0 (2.0–4.0) §†	3.0 (2.0–4.0) ‡¤
Pregravid body weight subsequent pregnancy (kg)	60.8 (55.0–70.0)	63.5 (56.6–73.8)	62.5 (55.8–70.0)	69.0 (59.0–77.3) ‡
Pregravid obesity subsequent pregnancy (*n*)	46 (9.3%)	13 (17.6%) ¶	10 (10.9%)	19 (19.0%) ‡
Pregravid overweight subsequent pregnancy (*n*)	125 (25.4%)	19 (25.7%)	22 (23.9%)	38 (38.0%) ‡•
Pregravid BMI subsequent pregnancy (kg/m^2^)	22.9 (20.8–25.9)	24.4 (21.7–27.6) ¶	23.5 (21.1–26.7)	26.2 (23.0–29.5) ‡•
Interpregnancy body weight change (kg)	2.0 (0.0–4.0)	3.0 (−0.4–7.5)	0.0 (−1.0–3.0) §†	0.0 (−1.0–5.0) ¤
Interpregnancy BMI change (kg/m^2^)	0.7 (0.0–1.7)	1.1 (−0.2–2.7)	0.0 (−0.4–1.2) §†	0.5 (−0.3–1.6) ¤
ISS High Risk for GDM in subsequent pregnancy (*n*)	46 (9.3%)	17 (23.0%) ¶	92 (100%) §†	100 (100%) ‡¤
Adherence to early OGTT subsequent pregnancy (*n*)	4 (8.7%)	6 (35.3%) ¶	52 (56.5%) §†	63 (63.0%) ‡
Early 75 g OGTT subsequent pregnancy, fasting glucose (mg/dL)	88.5 (87.8–89.0)	85.5 (81.5–93.3)	82.7 (79.0–87.0) §	94.0 (88.0–98.5) •
Early 75 g OGTT subsequent pregnancy, 1 h-PLG (mg/dL)	138.0 (135.0–141.8)	160.0 (147.8–170.8)	132.5 (115.3–146.3) †	154.5 (138.0–181.8) •
Early 75 g OGTT subsequent pregnancy, 2 h-PLG (mg/dL)	101.5 (99.5–107.0)	120.5 (109.8–144.0)	100.0 (91.8–115.0) †	126.5 (102.5–146.8) •
Early 75 g OGTT subsequent pregnancy, positive for GDM (*n*)	0 (0.0%)	3 (50.0%)	0 (0.0%)	52 (82.5%)
Standard 75 g OGTT subsequent pregnancy, gestational age (wg)	26.0 (25.0–27.0)	26.0 (26.0–27.0) ¶	26.0 (25.0–27.0)	26.0 (25.0–27.0)
Standard 75 g OGTT subsequent pregnancy, FG (mg/dL)	80.0 (76.8–84.0)	92.0 (84.5–95.3) ¶	82.0 (78.0–87.0) §†	92.0 (85.0–96.0) ‡•
Standard 75 g OGTT subsequent pregnancy, 1 h-PLG (mg/dL)	124.0 (104.0–144.0)	173.5 (141.0–186.3) ¶	142.0 (122.0–155.0) §†	184.0 (161.0–201.0) ‡¤•
Standard 75 g OGTT subsequent pregnancy, 2 h-PLG (mg/dL)	99.0 (88.0–113.0)	129.0 (108.5–146.0) ¶	107.0 (94.8–120.0) §†	154.0 (121.0–167.0) ‡¤•
Standard 75 g OGTT subsequent pregnancy, gestational weight gain (kg)	7.0 (5.0–9.0)	6.0 (5.0–9.0)	6.0 (3.0–9.0) §	6.0 (4.0–9.0)

Data are shown as medians (IQR) or *n* (%). The Mann–Whitney U test was used to determine significant differences in continuous variables between unpaired groups. The Fisher’s Exact test was used to compare proportions. The integral *p*-values are presented in Supplementary Table S1. ¶ *p* < 0.05 Group 2 vs. Group 1; § *p* < 0.05 Group 3 vs. Group 1; † *p* < 0.05 Group 3 vs. Group 2; ‡ *p* < 0.05 Group 4 vs. Group 1; ¤ *p* < 0.05 Group 4 vs. Group 2; • *p* < 0.05 Group 4 vs. Group 3. * denotes valid percentages. ISS: Italian “Istituto Superiore di Sanità”; OGTT: oral glucose tolerance test; FG: fasting glucose; PLG: post-load glucose; AUC: area under the curve; T2D: type 2 diabetes; wg: weeks of gestation. In Italy, ISS guidelines recommend early GDM screening at 16–18 wg for women with at least one of the following risk factors: (1) pregravid obesity; (2) pregravid and/or first trimester impaired fasting glucose (IFG, fasting plasma glucose levels of 100–125 mg/dL); or (3) previous GDM. High-risk women who test negative for GDM at this early screening are recommended to undergo another 75 g OGTT at standard 24–28 wg. GDM is diagnosed when one or more venous plasma glucose values exceed the IADPSG thresholds (fasting glucose ≥ 92 mg/dL, 1 h-PLG ≥ 180 mg/dL, and 2 h-PLG ≥ 153 mg/dL).

**Table 2:** Independent effects of 1-h and 2-h post-load glucose values in predicting new-onset GDM or GDM recurrence.

Regression Model	Parameter	Standardized β	Odds Ratio	Lower Bound (95% CI)	Upper Bound (95% CI)	*p* Value
New-onset GDM in subsequent pregnancy	Standard 75 g OGTT index pregnancy, 1 h-PLG (mg/dL) *	0.484	1.019	1.009	1.030	<0.001
Recurrent GDM in subsequent pregnancy	Standard 75 g OGTT index pregnancy, 2 h-PLG (mg/dL) *	0.762	1.028	1.002	1.054	0.035

* adjusted by including interpregnancy BMI gain, and pregravid BMI before the second pregnancy as covariates. CI: confidence interval.

**Table 3:** Assessment of 1-h and 2-h post-load glucose values above defined thresholds in predicting new-onset GDM or GDM recurrence.

Regression Model	Parameter	Standardized β	Odds Ratio	Lower Bound (95% CI)	Upper Bound (95% CI)	*p* Value
New-onset GDM in subsequent pregnancy	Standard 75 g OGTT index pregnancy 1 h-PLG ≥ 130 mg/dL *	0.914	2.496	1.507	4.133	<0.001
Recurrent GDM in subsequent pregnancy	Standard 75 g OGTT index pregnancy 2 h-PLG ≥ 153 mg/dL *	0.795	2.214	1.059	4.631	0.035

* adjusted by including interpregnancy BMI gain, and pregravid BMI before the second pregnancy as covariates.

## Data Availability

The data presented in this study are available upon request from the corresponding author due to ethical and privacy reasons.

## Supplementary Materials

The following supporting information can be downloaded at: https://www.mdpi.com/article/10.3390/jcm13175200/s1, Table S1: *p*-values for intergroup comparisons of clinical and 75 g OGTT characteristics of women.
