# Supplementary material for: Clinical Risk Factors and First Gestational 75 g OGTT May Predict Recurrent and New-Onset Gestational Diabetes in Multiparous Women
Source: J Clin Med. 2024 Sep 2;13(17):5200. doi: 10.3390/jcm13175200 (PMC11396485; doi:10.3390/jcm13175200)
Supplement: Supplementary file 1 [file jcm-13-05200-s001.zip › jcm-3149751-supplementary.pdf]

Table S1. ***P*** **values for intergroup comparisons of clinical and 75g OGTT characteristics of women.**

**Clinical and 75g OGTT characteristics of women**	**Group 2 vs. Group 1 ¶**	**Group 3 vs. Group 1 §**	**Group 4 vs. Group 1 ‡**	**Group 3 vs. Group 2 †**	**Group 4 vs. Group 2 ¤**	**Group 4 vs. Group 3 •**
Secondary or tertiary level education (n)	0.920	0.729	0.644	0.504	0.621	0.247
Smoker or Ex-smoker (n)	0.413	0.480	0.461	0.770	0.488	0.842
Familial history of T2D (n)	0.135	**0.009**	0.074	0.460	0.952	0.465
Age at menarche (y)	0.165	0.545	**0.017**	0.135	0.419	**0.022**
PCOS (n)	0.627	**<0.001**	**<0.001**	0.068	**<0.001**	**<0.001**
Nullipara women at index pregnancy (n)	0.597	0.101	0.166	0.427	0.566	0.083
Reproductive history of spontaneous abortion (n)	0.407	0.439	0.791	0.623	0.468	0.705
Pregravid bodyweight index pregnancy (kg)	0.173	0.070	**<0.001**	0.800	**0.012**	**0.013**
Pregravid BMI index pregnancy (kg/m^2)	0.059	0.095	**<0.001**	0.878	**0.005**	**0.002**
Pregravid obesity index pregnancy (n)	**0.044**	0.078	**<0.001**	0.795	0.376	0.222
Pregravid overweight index pregnancy (n)	0.839	0.432	**<0.001**	0.691	**0.008**	**0.016**
Maternal age index pregnancy (y)	0.227	**0.003**	0.135	0.204	0.905	0.242
Standard 75g OGTT index pregnancy, gestational age (wg)	0.307	**0.008**	0.925	**0.008**	0.385	0.065
Standard 75g OGTT index pregnancy, FG (mg/dL)	**0.002**	**<0.001**	**<0.001**	**<0.001**	**0.001**	**0.044**
Standard 75g OGTT index pregnancy, 1h-PLG (mg/dL)	**<0.001**	**<0.001**	**<0.001**	**<0.001**	**0.001**	0.082
Standard 75g OGTT index pregnancy, 2h-PLG (mg/dL)	**0.033**	**<0.001**	**<0.001**	**<0.001**	**0.001**	0.114
Standard 75g OGTT index pregnancy, AUC glucose (mg*h/dL)	**<0.001**	**<0.001**	**<0.001**	**<0.001**	**0.001**	**0.028**
Standard 75g OGTT index pregnancy, gestational weight gain (kg)	0.685	0.250	**<0.001**	0.591	0.051	0.097
Gestational weigh gain at the last follow-up before delivery (kg)	_	_	_	_	_	0.052
Insulin therapy index pregnancy (n)	_	_	_	_	_	**0.012**
Macrosomic birth index pregnancy (n)	0.346	1.000	0.430	0.445	1.000	1.000
Preterm birth index pregnancy (n)	**0.032**	**0.003**	0.507	0.733	0.313	0.106
Maternal age subsequent pregnancy (y)	0.116	**0.021**	0.501	0.612	0.428	0.242
Interpregnancy age change (y)	0.241	**<0.001**	**0.027**	**<0.001**	**0.013**	0.399
Pregravid bodyweight subsequent pregnancy (kg)	0.082	0.406	**0.023**	0.393	0.539	0.132
Pregravid obesity subsequent pregnancy (n)	**0.030**	0.645	**0.005**	0.214	0.809	0.116
Pregravid overweight subsequent pregnancy (n)	0.953	0.770	**0.010**	0.794	0.087	**0.035**
Pregravid BMI subsequent pregnancy (kg/m^2)	**0.024**	0.608	**0.008**	0.149	0.524	**0.039**
Interpregnancy bodyweight change (kg)	0.074	**0.005**	0.212	**0.003**	**0.041**	0.249
Interpregnancy BMI change (kg/m^2)	0.075	**0.006**	0.171	**0.003**	**0.033**	0.292
ISS High Risk for GDM at subsequent pregnancy (n)	**<0.001**	**<0.001**	**<0.001**	**<0.001**	**<0.001**	_
Adherence to early OGTT subsequent pregnancy (n)	**0.016**	**<0.001**	**<0.001**	**0.047**	0.012	0.360
Early 75g OGTT subsequent pregnancy, fasting glucose (mg/dL)	0.668	**0.038**	0.121	0.306	0.101	**<0.001**
Early 75g OGTT subsequent pregnancy, 1h-PLG (mg/dL)	0.171	0.535	0.162	**0.014**	0.966	**<0.001**
Early 75g OGTT subsequent pregnancy, 2h-PLG (mg/dL)	0.087	0.702	0.150	**0.016**	0.820	**<0.001**
Early 75g OGTT subsequent pregnancy, positive for GDM (n)	_	_	_	_	0.093	_
Standard 75g OGTT subsequent pregnancy, gestational age (wg)	**0.007**	0.861	0.070	0.069	0.810	0.209
Standard 75g OGTT subsequent pregnancy, FG (mg/dL)	**<0.001**	**0.006**	**<0.001**	**<0.001**	0.672	**<0.001**
Standard 75g OGTT subsequent pregnancy, 1h-PLG (mg/dL)	**<0.001**	**<0.001**	**<0.001**	**<0.001**	**0.018**	**<0.001**
Standard 75g OGTT subsequent pregnancy, 2h-PLG (mg/dL)	**<0.001**	**0.002**	**<0.001**	**<0.001**	**<0.001**	**<0.001**
Standard 75g OGTT subsequent pregnancy, gestational weight gain (kg)	0.270	**0.030**	0.372	0.432	0.854	0.549
